# Association Between Higher Plasma Lutein, Zeaxanthin, and Vitamin C Concentrations and Longer Telomere Length: Results of the Austrian Stroke Prevention Study

**DOI:** 10.1111/jgs.12644

**Published:** 2014-01-15

**Authors:** Abhijit Sen, Gunther Marsche, Paul Freudenberger, Michael Schallert, Anna M Toeglhofer, Christoph Nagl, Reinhold Schmidt, Lenore J Launer, Helena Schmidt

**Affiliations:** * Research Unit for Genetic Epidemiology, Institute of Molecular Biology and Biochemistry, Center of Molecular Medicine, Medical University of GrazGraz, Austria; † Institute of Experimental and Clinical Pharmacology, Medical University of GrazGraz, Austria; ‡ Department of Neurology, Medical University of GrazGraz, Austria; § Laboratory of Epidemiology and Population Sciences, National Institute on AgingBethesda, Maryland

**Keywords:** telomere length, vitamin C, lutein zeaxanthin, aging, antioxidants, oxidative stress

## Abstract

**Objectives:**

To examine the association between plasma concentrations of antioxidative micronutrients and leukocyte telomere length (LTL) in elderly adults.

**Design:**

Cross-sectional cohort study.

**Setting:**

Austrian Stroke Prevention Study, a population-based cohort study on brain aging.

**Participants:**

Individuals with a mean age of 66 ± 7 (n = 786; 58% female).

**Measurements:**

Concentrations of vitamin C, lutein, zeaxanthin, *β*-cryptoxanthin, canthaxanthin, lycopene, *α*- and *γ*-tocopherol, *α*- and *β*-carotene, and retinol in plasma, advanced oxidation protein products as a measure of oxidative stress in serum, and LTL were measured. Vitamins and carotenoids were measured using high-performance liquid chromatography, advanced oxidation protein products using spectrophotometry, and telomere length using quantitative real-time polymerase chain reaction.

**Results:**

Multiple linear regression analyses with adjustment for age and sex demonstrated that higher lutein, zeaxanthin, and vitamin C concentrations were strongly associated with longer telomere length. The associations were independent of body mass index, maximum oxygen uptake, and vascular risk factors and were not mediated by advanced oxidation protein products content.

**Conclusion:**

This study provides first evidence that higher lutein, zeaxanthin, and vitamin C concentrations in plasma are associated with longer LTL in normal elderly persons and suggest a protective role of these vitamins in telomere maintenance.

Progressive shortening of telomere length with each cell division is associated with cellular senescence and apoptosis.^1^ Shorter leukocyte telomere length (LTL) is linked to aging^2^,^3^ and age-related diseases such as cardiac disease,^4^,^5^ diabetes mellitus,^6^,^7^ hypertension,^8^,^7^ increased cancer,^9^ and mortality.^10^ Heritability of LTL ranges from 36% to 84% depending on population.^11^ Environmental and lifestyle factors such as body mass index (BMI),^5^,^12^ smoking,^12^ socioeconomic status,^13^ and dietary habits^14^ influence LTL. Shorter LTL is associated with oxidative stress.^15^,^16^ Evidence from previous in vitro,^16^ in vivo,^17^ and clinical studies^4^,^7^,^18^ suggests that an imbalance between free oxygen radicals and antioxidant concentration in the cellular environment contributes to telomere attrition. Epidemiological studies report an inverse association between dietary micronutrients and oxidative stress,^19^ which suggests that dietary micronutrients may exert protective effects on telomere shortening through antioxidative properties. A limited number of studies have investigated the association between dietary antioxidants and LTL by administering self-reported questionnaires.^14^,^20^ To the knowledge of the authors of the current study, no study has investigated the association between directly measured plasma levels of antioxidative micronutrients and LTL. The current study was designed to test the hypothesis that higher plasma concentrations of vitamin C, lutein and zeaxanthin (Lu∼Zx), *β*-cryptoxanthin, canthaxanthin, lycopene, *α*- and *γ*-tocopherol, *α*- and *β*-carotene, and retinol are associated with longer LTL. In addition to assessment of the independent effect of individual micronutrients on LTL, the pooled effects of micronutrients in the provitamin A, non-provitamin A, and vitamin E subgroups and total antioxidant status were evaluated. Whether low oxidative stress, as measured according to advanced oxidation protein product (AOPP) content in serum, mediated a favorable effect of the micronutrients on LTL was further explored.^21^ The study was performed in 786 participants of the Austrian Stroke Prevention Study (ASPS), a population-based cohort study in elderly adults.

## Subjects and Methods

### Study Design and Recruitment

**Figure 1:**
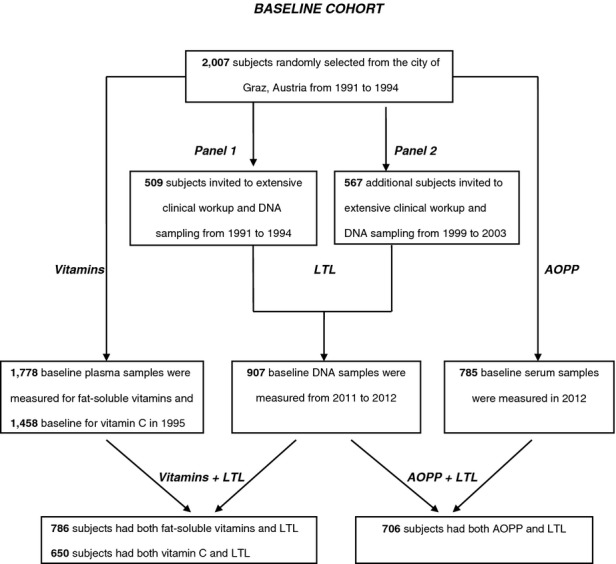
The flowchart illustrates the study design and recruitment of participants aged 46 to 85 into the current study. In Panel 1 (baseline visit), recruited participants underwent extensive clinical examination, including neuroimaging; additional participants included in Panel 2 (baseline visit) underwent the same extensive clinical examination. Deoxyribonucleic acid (DNA), serum, and plasma samples collected at baseline examination were available for leukocyte telomere length (LTL), advanced oxidation protein product (AOPP) content, and vitamin measurements, respectively. Individuals with vitamin and LTL measurement were included (N = 786). Vitamins (fat-soluble) = lutein, zeaxanthin, *β*-cryptoxanthin, canthaxanthin, lycopene, *α*- and *γ*- tocopherol, *α*- and *β*-carotene, and retinol.

Details of the ASPS have been published previously.^22^ The institutional review board of the Medical University of Graz approved the study. Figure1 shows the algorithm of recruitment into the current study. In brief, 2007 individuals aged 45 to 86 were recruited from the official registry of residents of city of Graz, Austria, between 1991 and 1994. Individuals were excluded from the study if they had a history of neuropsychiatric disease, including previous cerebrovascular attacks and dementia, or an abnormal neurological examination determined on the basis of a structured clinic interview and a physical and neurological examination. All subjects were ambulatory. Plasma antioxidative vitamins were measured in 1995 from samples collected at the baseline examination. One thousand seven hundred subjects had measurements of fat-soluble antioxidative micronutrients and 1,458 of vitamin C. Every fourth participant (n = 509) of the original cohort was included in an extensive clinical examination, including neuroimaging and deoxyribonucleic acid (DNA) sampling between 1991 and 1994 (Panel 1, baseline visit). Between 1999 and 2003, a second panel of 567 subjects from the same source population as in Panel 1 was invited to participate in the same extensive diagnostic examination (Panel 2, baseline visit). Subjects from the first and second panels were pooled, resulting in 1,076 subjects, from whom DNA samples for LTL measurement were available in 907. The present study included those 786 ASPS participants, who had both antioxidative micronutrients and LTL measurements.

### Laboratory Assays

#### DNA Plating and LTL Measurement

DNA samples were extracted from whole blood collected in ethylenediaminetetraacetic acid (EDTA) tubes using the phenol–chloroform method, quantified using spectrophotometry, and checked for degradation using agarose gel electrophoresis. All DNA samples were frozen without delay upon extraction and stored at −80°C constantly. Two quantitative polymerase chain reactions (PCRs), one for telomere repeat copy (‘T’) and one for a single-copy gene (*36B4,* ‘S’), were prepared for each sample. In a total volume of 6.5 μL, 2 μL of DNA (2.5 ng/μL) was plated with 4.5 μL of PCR master mix using an automated eight-channel pipetting robot (JANUS, PerkinElmer, Waltham, MA). All reactions for ‘T’ and ‘S’ amplifications were performed in quadruplicate in 384-well plates. The primers for ‘T’ and ‘S’ were designed as previously described.^23^ In addition, triplicates of no-template control, pooled reference DNA and five serially diluted standards were included on each plate.^24^ Quantitative real-time PCR was done on a 7900 HT Fast Real-Time PCR cycler (Applied Biosystems, Foster City, CA). The same technician assayed samples under identical conditions using standard operational protocols. The technician was blinded to the clinical and laboratory data of the participants. Intra-assay coefficients of variability (%) for ‘T’ and ‘S’ reaction were 0.25 and 0.22, respectively. Interassay coefficients of variability (%) for ‘T’ and ‘S’ reaction were 3.03 and 2.49, respectively.

### Calculation of LTL

The relative LTL (T/S ratio) was calculated according to Cawthon's modified method^23^,^24^ and normalized to the reference DNA pooled from 24 subjects, including 50% women (mean age 51.5). In the current analysis, the skewed distribution of LTL was natural logarithm transformed, and the *z*-score was calculated by standardizing the LTL in comparison to the mean. Correlation between LTL measured using quantitative real-time PCR and the southern blot method (*TeloTAGGG* Telomere Length Assay kit, Roche, Mannheim, Germany) was also tested in 20 individuals. The correlation coefficient between the two methods was 0.61.

### Measurement of Carotenoids and Vitamins

The blood sampling method for antioxidant measurements has been described previously.^25^ Ten mL of blood was collected into EDTA-containing plastic tubes and centrifuged. Two 2-mL plasma samples were transferred to Eppendorf tubes, and a third 0.5-mL plasma sample was pipetted into plastic tubes containing 0.5 mL of 10% aqueous metaphosphoric acid. The tubes were promptly frozen and stored at −70°C. The time between blood sampling and the start of freezing of plasma and acidified plasma varied from 45 minutes to 2 hours. Storage until analysis varied from 1 month to 2 years. For measurements of lipophilic antioxidants, the deep-frozen plasma tubes were thawed and then extracted within 30 minutes after thawing. Plasma (0.4 mL), water (0.4 mL, vortexed), ethanol with 0.1% butylated hydroxytoluene (0.8 mL, vortexed 10 seconds), and hexane (2 mL, stored over bidistilled water containing 20 μmol/L EDTA) were successively pipetted into 10-mL Pyrex tubes. After this mixture was vortexed for 1 minute, it was centrifuged. A volume of 1.2 mL of the upper hexane phase was transferred into brown crimp vials and dried in a speed-vac for 10 minutes at room temperature. The residue was dissolved in 0.3 mL ethanol–ethyl acetate in the ratio of 10:1, and 20 μL was injected into the high-performance liquid chromatography (HPLC) apparatus (5 μm, 125 × 4 mm, LiChrospher 100 RP18, Merck Millipore Billerica, MA). Separation was performed in isocratic mode with a mixture of acetonitrile, methanol, ethanol, water (50/60/20/2) containing 0.01% ammonium acetate (flow rate 1.2 mL/min). The effluent was monitored using two detectors in series; an ultraviolet-vis detector was set to 450 nm for detection of carotenoids, and a fluorescence detector set initially to 325/500 nm for detection of retinol and after 3.8 minutes to 292/335 nm for detection of tocopherols. For measurement of ascorbate, the deep-frozen tubes with the metaphosphoric acid plasma were thawed and centrifuged at 9,000 revolutions per minute. A volume of 0.1 mL of the supernatant was mixed with 0.4 mL of HPLC-eluant, centrifuged, and injected by the autosampler into the HPLC (LiChrospher 100 RP18, 5 μm, 250 × 4 mm). The HPLC-eluant was prepared by adding 4.3 mL of 70% perchloric acid and 100 mg of EDTA to 1 L of bidistilled water. Flow rate was 1 mL/min. The effluent was monitored using an electrochemical detector set to 0.6V against a silver–silver chloride reference electrode filled with 3 mol/L of lithium chloride. Peak quantification was performed with at least two standard mixtures of ascorbic acid. The time between thawing and HPLC separation did not exceed 3 hours. Range of plasma antioxidants concentrations in the present study was similar to those in various other studies, including the Third National Health and Nutrition Examination Survey.^26^

#### Measurement of Serum AOPP Content

Serum for AOPP measurement was available in 785 subjects. Lipoproteins in serum were precipitated by adding magnesium chloride (1 μL of a 1 mol/L stock solution) and phosphotungstate (2 μL of a 4% stock solution in 0.19 mol/L sodium hydroxide) to 20 μL of serum and incubated for 20 minutes. Samples were subsequently centrifuged at 1,000 *g* for 20 minutes, and the supernatants were carefully removed. AOPP content was immediately measured in the supernatants at 340 nm under acidic conditions (containing 10% acetic acid v/v) using a spectrophotometer and expressed as chloramine-T equivalents.^27^

### Statistical Analysis

Statistical analysis was performed using SPSS version 19 (IBM, Ehningen, Germany). Normal distribution of the quantitative variables was assessed using the Kolmogorov-Smirnov test and Q-Q plots. All quantitative variables except LTL and *α*- and *β*-carotene were normally distributed. The skewed distribution of the dependent variable LTL was natural logarithm transformed, and a *z*-score was calculated by subtracting the group mean from the individual's value and dividing the result by the standard deviation. *Z*-scores were similarly calculated for each antioxidative micronutrient. Composite *z*-scores were constructed for the subgroups of provitamin A (*β*-cryptoxanthin, *α*- and *β*-carotene, retinol), non-provitamin A (Lu∼Zx, lycopene, canthaxanthin), and vitamin E (*α*- and *γ*-tocopherol) by taking the mean of the individual nutrient *z*-scores per subgroup. The total antioxidant status of all micronutrients was calculated by summing the *z*-scores and dividing by the number of all antioxidants. To see whether previously reported findings could be replicated, the associations between z(lnLTL) and age, sex, diabetes mellitus, hypertension, smoking, cardiac disease, BMI, cardiorespiratory fitness measured as maximal oxygen consumption (VO_2_max), and C-reactive protein (CRP) were tested using multiple linear regression and adjusting for age and sex when appropriate.

Multiple linear regression models were used to estimate the effect of the individual micronutrients, the fat-soluble micronutrient subgroups and the total antioxidant status of micronutrients on telomere length. Three models with different sets of covariates were used. In Model 1, age and sex were adjusted for; in Model 2 BMI and VO_2_max were additionally adjusted for; and in Model 3, all model 2 variables plus vascular risk factors including alcohol consumption, smoking status, hypertension, and diabetes mellitus were adjusted for.

A previously developed bootstrapping procedure^28^ (available for download from http://www.afhayes.com/spss-sas-and-mplus-macros-and-code.html) was used to test the extent to which AOPP content mediated the association between Lu∼Zx and z(lnLTL) and between vitamin C and z(lnLTL). A quantitative approach was taken to assess mediation (Figure S1). The association between variables A (micronutrients) and C (LTL) was tested when a variable B2 (AOPP) was added to the model that also included confounding variables B1 (BMI, VO_2_max, and vascular risk factors). The model specifically generated estimates of the association between the dependent (C) and independent (A) variables, the association between the independent variable (A) and the mediator (B2), and the association between the mediator (B2) and the dependent variable (C). There is a formal test of whether B2 is a significant mediator of the association between C and A. Thus, this approach provides information on the mediation of B2, as well as the direct independent association between B2 and A and between B2 and C. The bootstrapping method was then used to calculate 95% confidence intervals (CIs) for the indirect effects by repeating the sampling procedure one thousand times. Secondary analyses were conducted on nutrients that were significantly associated with z(lnLTL). Interactions between these nutrients and age, sex, smoking, hypertension, and BMI were tested using cross-product terms. Finally, the quartile distribution of nutrients significantly related to LTL was assessed, and the median of LTL of the lowest and the highest quartile was compared using a *t*-test.

## Results

The mean age of the current cohort was 66 ± 7. There were 456 (58%) women. Assessment and definitions of risk factors and VO_2_max have been published previously.^22^,^29^ Table1 describes the distribution of demographic characteristics, BMI, VO_2_max, vascular risk factors, antioxidative micronutrients, oxidative stress marker, and LTL in the cohort.

**Table 1:** Characteristics of Participants from the Austrian Stroke Prevention Study (ASPS) Who Provided DNA Samples for Measurement of Relative Leukocyte Telomere Length (LTL)

Characteristic	Total, N = 786	Female, n = 456	Male, n = 330	<65, n = 374	≥65, n = 412
Demographic, mean ± SD					
Age	66.0 ± 7.0	66.5 ± 8.0^b^	65.2 ± 7.6	59.1 ± 4.3^c^	72.2 ± 4.7
Body mass index, kg/m^2^	26.9 ± 4.1	26.7 ± 4.6	27.2 ± 3.2	26.3 ± 3.9^c^	27.4 ± 4.1
Maximum oxygen uptake	1.96 ± 0.46	1.75 ± 0.36	2.20 ± 0.44	2.06 ± 0.46	1.86 ± 0.42
Vascular risk factor					
Alcohol consumption, n (%)	87 (11)	17 (3.7)^c^	70 (21)	44 (11.7)	43 (10.4)
Former smoker, n (%)	220 (28)	72 (16)^c^	148 (45)	107 (29)	114 (28)
Current smoker, n (%)	84 (11)	31 (7)^c^	53 (16)	49 (13)	35 (9)
Diabetes mellitus, n (%)	76 (10)	41 (9)	35 (10.6)	22 (5.9)	54 (13)
Fasting glucose, mg/dL, mean ± SD	95.3 ± 24.6	94.2 ± 25.3	96.8 ± 23.5	93.3 ± 22.1^b^	97.1 ± 26.6
Glycosylated hemoglobin, % mean ± SD	5.7 ± 0.75	5.8 ± 0.76	5.7 ± 0.74	5.6 ± 0.72^c^	5.9 ± 0.76
Insulin resistance, mean ± SD	9.2 ± 5.4	9.4 ± 6.2	9.1 ± 4.4	8.9 ± 5.4	10.1 ± 5.6
Hypertension, n (%)	574 (73)	331 (72)	243 (73.6)	224 (60)	350 (85)
Mean systolic blood pressure, mm/Hg, mean ± SD	145.0 ± 23.8	145.5 ± 24.9	144.8 ± 22	137.2 ± 20.8^c^	152.2 ± 24.1
Mean diastolic blood pressure, mm/Hg, mean ± SD	88.2 ± 10.6	88.4 ± 10.9	88.0 ± 10.1	86.6 ± 10^c^	89.7 ± 10.9
C-reactive protein, mg/L, median (IQR)	2.0 (0.9–4.0)	2.2 (0.9–4.4)	1.7 (0.8–3.3)	1.9 (0.9–3.4)	2.1 (0.9–4.5)
Antioxidants, μmol/L					
Provitamin A: carotenoids					
*α*-carotene, median (IQR)	0.07 (0.04–0.12)	0.08 (0.05–0.13)	0.05 (0.03–0.08)	0.07 (0.04–0.12)	0.07 (0.04–0.11)
*β*-carotene, median (IQR)	0.40 (0.25–0.67)	0.49 (0.32–0.78)	0.31 (0.19–0.48)	0.40 (0.25–0.71)	0.39 (0.25–0.64)
*β*-cryptoxanthin, mean ± SD	0.26 ± 0.23	0.30 ± 0.24^c^	0.22 ± 0.21	0.26 ± 0.23	0.26 ± 0.22
Retinol, mean ± SD	1.93 ± 0.66	1.79 ± 0.63^c^	2.13 ± 0.65	1.91 ± 0.70^b^	1.84 ± 0.61
Non-provitamin A: carotenoids, mean ± SD					
Lutein and zeaxanthin	0.56 ± 0.23	0.57 ± 0.24	0.54 ± 0.22	0.56 ± 0.22	0.56 ± 0.23
Canthaxanthin	0.12 ± 0.06	0.12 ± 0.06^c^	0.11 ± 0.06	0.12 ± 0.06^b^	0.11 ± 0.06
Lycopene	0.22 ± 0.17	0.22 ± 0.18	0.21 ± 0.17	0.24 ± 0.18^b^	0.19 ± 0.16
Vitamin E, mean ± SD					
*α*-tocopherol	30.1 ± 8.9	30.6 ± 8.7^b^	29.4 ± 9.3	30.1 ± 9.0	30.2 ± 8.9
*γ*-tocopherol	2.30 ± 1.09	2.24 ± 0.98^b^	2.45 ± 1.20	2.31 ± 1.10	2.34 ± 1.10
Vitamin C	57.7 ± 20.5	63.6 ± 18.9^c^	49.5 ± 19.8	58.7 ± 20.5	56.8 ± 20.5
Advanced oxidation protein product, nmol/mg of protein, mean ± SD	3.3 ± 2.6	3.1 ± 2.4^c^	3.6 ± 2.7	3.5 ± 2.9	3.1 ± 2.2
LTL, median (IQR)^a^	0.61 (0.05–2.60)	0.60 (0.05–2.60)	0.62 (0.23–2.37)	0.62 (0.16–2.23)	0.60 (0.05–2.60)

The unpaired *t*-test was performed for normally distributed continuous variables, the chi-square test for categorized variables, and the Mann–Whitney *U*-test for nonnormally distributed data.

SD = standard deviation; IQR = interquartile range.

a Calculated relative LTL was normalized to a T/S ratio of reference deoxyribonucleic acid (pooled from 24 subjects of different age and sex).

b *P*< .05.

c *P*< .001.

The associations between z(lnLTL) and age, sex, diabetes mellitus, hypertension, smoking status, cardiac disease, BMI, and the inflammatory marker CRP are presented in supplementary Table S1.

Age and BMI were the only factors positively and significantly related to z(lnLTL). Table2 shows the independent effect of each micronutrient, of fat-soluble micronutrient subgroups, and of total antioxidant status on LTL. Of all micronutrients, Lu∼Zx and vitamin C remained significantly and independently associated with LTL when adjusted for age and sex (Model 1) (Lu∼Zx: *β* = 0.079, *P*= .03; vitamin C: *β* = 0.160, *P*< .001). After additional adjustment for BMI and VO_2_max in Model 2, the relationship between Lu∼Zx and LTL became stronger (*β* = 0.117, *P*= .01), whereas the association between vitamin C and LTL remained virtually unchanged (*β*= 0.152, *P*= .004). By adding each factor individually to the model, it was found that VO_2_max was responsible for observed effect (data not shown). Further adjustment for vascular risk factors did not change effect sizes (Model 3) (Lu∼Zx: *β* = 0.120, *P*= .006; vitamin C: *β*= 0.146, *P*= .004). The effects of Lu∼Zx and of vitamin C on LTL were stronger in men than in women. For Lu∼Zx, the association in men was significant (*β*= 0.148, *P*= .02), but it was nonsignificant in women (*β* = 0.070, *P*= .25). For vitamin C, the relationship was again significant in men (*β*= 0.166, *P*= .02), whereas in women, there was a nonsignificant trend (*β* = 0.123, *P*= .07).

**Table 2:** Multiple Linear Regression Analysis to Explore Relationship Between Antioxidative Micronutrients and z(lnLTL) in 786 Individuals

	Model 1	Model 2	Model 3
Variables	*β*	*β*	*β*
Individual micronutrient *z*-scores			
Lutein and zeaxanthin	0.079^b^	0.107^c^	0.120^c^
*β*-cryptoxanthin	0.060	0.041	0.040
Canthaxanthin	0.018	0.049	0.056
Lycopene	−0.080^b^	−0.064	−0.069
*α*-carotene	−0.027	−0.020	−0.028
*β*-carotene	−0.069	−0.065	−0.077
*α*-tocopherol	−0.012	0.013	0.019
*γ*-tocopherol	−0.002	0.041	0.044
Vitamin C^a^	0.160[Table-fn tf2-5]	0.152^c^	0.146^c^
Retinol	−0.055	−0.065	−0.058
Subgroup micronutrient *z*-scores			
Provitamin A	−0.063	−0.063	−0.068
Non-provitamin A	0.003	0.033	0.039
Vitamin E	−0.006	0.038	0.043
Total antioxidant status	−0.014	0.044	0.037

Non-provitamin A: sum of lutein, zeaxanthin, lycopene, and canthaxanthin *z*-scores divided by number of antioxidants. Provitamin A: sum of *α*- and *β*-carotene, *β*-cryptoxanthin, and retinol *z*-scores divided by number of antioxidants. Vitamin E: sum of *α*-tocopherol and *γ*-tocopherol *z*-scores divided by number of antioxidants. Total antioxidant status: sum of all antioxidative *z*-scores divided by number of antioxidants. Model 1 adjusted for age and sex. Model 2 adjusted for factors in Model 1, body mass index, maximum oxygen uptake. Model 3 adjusted for factors in Model 2, alcohol consumption, smoking, diabetes mellitus, and hypertension.

a The 650 individuals had leukocyte telomere length (LTL) and vitamin C measurement.

b *P*< .05.

c *P*< .01.

d *P*< .001.

Bootstrapping demonstrated that the indirect effect of Lu∼Zx (*β*′ = 0.0009, 95% CI = −0.0110 to 0.0288) and vitamin C (*β*′ = 0.0001, 95% CI = −0.0006 to 0.0002) on LTL through AOPP content, was not statistically significant (Table S2). There was also no statistically significant effect of the interaction between age, sex, smoking, hypertension, or BMI and Lu∼Zx and vitamin C on LTL (data not shown). It was also observed that study participants with a plasma Lu∼Zx concentration in the lowest quartile had 5% shorter telomere length than their counterparts in the highest quartile (*P*= .05 for linear trend). For vitamin C, this difference was 10% (*P*= .04 for linear trend). Women and men in the lowest quartile of plasma Lu∼Zx had 10.5% and 7% shorter telomere length, respectively, than those in the highest quartile. For vitamin C, these figures were 21% and 41%, respectively.

## Discussion

### Principal Findings

This is the first study to investigate the association between plasma levels of antioxidative micronutrients such as Lu∼Zx, vitamin C, *β*-cryptoxanthin, canthaxanthin, lycopene, *α*- and *γ*-tocopherol, *α*- and *β*-carotene, and retinol and LTL in a population-based elderly cohort. A strong and highly significant protective effect of Lu∼Zx and vitamin C on LTL was found. No other antioxidants or the pooled subgroups of provitamin A, non-provitamin A, vitamin E, and total antioxidant status of all micronutrients were associated with telomere length. The proportion of LTL variability that was explained by Lu∼Zx was 0.6%, and for vitamin C, it was 4%. It was observed that serum AOPP content did not mediate the effects of Lu∼Zx and vitamin C on LTL.

### Results in Relation to Other Studies

Several studies have reported that eating fruits and vegetables rich in vitamin C and Lu∼Zx reduce the incidence of age-related chronic disease^30^,^31^ and total mortality.^32^,^33^ Other studies have demonstrated that LTL is associated with survival^10^ and chronic diseases.^5^,^6^,^8^,^7^ The current study investigated whether antioxidant status modified LTL. The findings are consistent with those of the cross-sectional Sisters Study of 586 middle aged to elderly women. The authors reported that higher intake of vitamin C from foods and multivitamin supplements was associated with longer telomere length,^20^ but another study found no association between plasma vitamin C and LTL in a homogeneous population of 4,441 middle aged to elderly women.^34^ The fact that the first study included only women might have been responsible for the discrepant findings between that study^20^ and the current one, which included men and women. Consistent with the previous study's findings, the sex-stratified analysis in the current investigation also showed that the association between vitamin C concentration and LTL was weaker in women. A significant association was observed only in men. The same was true for the relationship between Lu∼Zx and LTL. A relationship between vitamin D and LTL has also been reported.^35^ Higher concentrations of vitamin D were associated with longer telomere length in a study of 2,160 women aged 18 to 79 from a large population-based cohort of twins. Vitamin D was not measured in the current study cohort.

### Possible Mechanisms

#### Antioxidative Properties of Lu∼Zx and Vitamin C

Lu∼Zx and vitamin C may protect LTL through their antioxidative properties by improving intracellular redox status. Oxidative stress is considered to be one of the major causes of DNA damage and LTL shortening. Several in vitro and in vivo studies have shown that Lu∼Zx^36^,^37^ and vitamin C^38^ prevent DNA breakage and modulate DNA repair through their antioxidative activity. Studies have also shown that the antioxidative action of vitamin C protects telomeres from attrition in cell culture.^39^ In the context of the current study findings, previous in vitro, in vivo, and clinical studies suggested that non-provitamin A carotenoids have greater protective effects against DNA damage than provitamin A carotenoids.^37^ This is consistent with the finding of the current study that lutein and zeaxanthin, which are non-provitamin A carotenoids, were directly related to LTL, whereas no such association was seen for the provitamin A carotenoids retinol, *β*-carotene, *α*-carotene, and *β*-cryptoxanthin.

#### Indirect Mechanisms of Lu∼Zx and Vitamin C

Other mechanistic properties by which Lu∼Zx and vitamin C may slow telomere attrition are immune-modulatory actions,^40^,^41^ anti-inflammatory activity,^42^,^43^ modulation of apoptosis,^44^,^45^ and lymphocyte proliferation.^46^ Of particular interest are data demonstrating that saturated vitamin C levels in endothelial cells are necessary to protect tetrahydrobiopterin from oxidation and to provide optimal conditions for cellular nitric oxide synthesis.^47^ Endothelium-derived nitric oxide is a potent signaling molecule in the cardiovascular system, participating in many processes such as vascular relaxation, inhibition of platelet aggregation, and preservation of normal vessel wall structure.^48^ Endothelial nitric oxide synthase activity has been reported to regulate telomerase activity and delay endothelial cell senescence.^49^,^50^

#### Healthy Lifestyle

Higher Lu∼Zx and vitamin C levels may be a marker of a healthy lifestyle. Therefore, their association with LTL may solely reflect the protective role of healthy lifestyle on biological aging.^33^,^51^

### Strength and Limitations

There are several limitations of the current study. First, the measurement of micronutrients, LTL, and AOPP content at a single time point limits causal inferences. Further insights can be expected from longitudinal studies. Second, AOPP content was measured as a single marker of oxidative stress. It is conceivable that a wider panel of lipid and especially DNA oxidative stress markers, such as urinary 8-hydroxy deoxyguanosine, would provide more information on possible oxidative stress-related actions of Lu∼Zx and vitamin C on LTL. Third, although multiple confounders were adjusted for, the possibility that residual confounding may at least partially explain the findings cannot be fully excluded. This study has several strengths. Actual plasma concentrations of carotenoids and vitamins were used, in contrast to information based on self-reported questionnaires.^14^,^20^ This approach should have resulted in less bias and imprecision than with self-reported dietary data. Another strength of the study is its large sample size and the extensive diagnostic examination of study participants. All DNA, plasma, and serum samples were obtained in a standardized fashion and were frozen at −80°C before measurements. Measuring each DNA sample in quadruplicate, the low intra- and interassay variability, and the established correlation (*r*= 0.61) between the real-time PCR and the southern blot method ensured the quality of the LTL measurements. Previously reported inverse associations between age and LTL were replicated.^2^,^3^ Mixed results were obtained for BMI,^4^,^12^ hypertension,^8^,^7^,^14^ diabetes mellitus,^4^,^6^ cardiac disease,^4^,^5^ smoking,^4^,^12^,^34^ and high-sensitivity CRP.^4^,^13^ In line with previous findings, inverse but statistically nonsignificant associations were found between diabetes mellitus, hypertension, cardiac disease, CRP level, and LTL.

## Conclusion and Implications

The findings of a strong association between the antioxidative micronutrients Lu∼Zx, vitamin C, and LTL may have important preventive implications. LTL shortening is associated with advancing age and with age-related diseases such as stroke, diabetes mellitus, cardiovascular diseases, and cancer. If these associations are causal, one might assume that Lu∼Zx– and vitamin C–related LTL protection has the potential to prevent or modify the course of numerous widespread diseases that are among the major contributors to mortality and morbidity in aging societies.

## Supporting Information

**Table S1.** Association Between z(lnLTL) with Age, Sex and Cardiovascular Disease Risk Factors: Austrian Stroke Prevention Study.**Table S2.** Association of Antioxidative Micronutrients on Telomere Length, Mediated by Serum AOPP. **Figure S1.** Model to test the mediating effect of variables (B1 = confounders) and (B2 = mediator) on the association of independent (A = antioxidative micronutrient represented in *z*-scores) and dependent (C = telomere length represented in natural logarithmic transformed *z*-scores) variables where path *β*AB1: effect estimate of predictor(s) to confounder(s) *β*AB2: effect estimate of predictor(s) to mediator; *β*B1C: effect estimate of confounder(s) to dependent variable; *β*B2C: effect estimate of mediator to dependent variable; dotted line represents c path = adjusted for confounders and c' path = in addition to confounders also adjusted for mediator. Confounders = age, sex, alcohol consumption, body mass index, smoking status, maximum oxygen uptake, hypertension and diabetes status. Mediator = serum advanced oxidation protein product content.
