# Supplementary material for: Association Between Higher Plasma Lutein, Zeaxanthin, and Vitamin C Concentrations and Longer Telomere Length: Results of the Austrian Stroke Prevention Study
Source: J Am Geriatr Soc. 2014 Jan 15;62(2):222–9. doi: 10.1111/jgs.12644 (PMC4234001; doi:10.1111/jgs.12644)
Supplement: Supplementary file 1 — Table S1. Association Between z(lnLTL) with Age, Sex and Cardiovascular Disease Risk Factors: Austrian Stroke Prevention Study.Table S2. Association of Antioxidative Micronutrients on Telomere Length, Mediated by Serum AOPP. Figure S1. Model to test the mediating effect of variables (B1 = confounders) and (B2 = mediator) on the association of independent (A = antioxidative micronutrient represented in z-scores) and dependent (C = telomere length represented in natural logarithmic transformed z-scores) variables where path βAB1: effect estimate of predictor(s) to confounder(s) βAB2: effect estimate of predictor(s) to mediator; βB1C: effect estimate of confounder(s) to dependent variable; βB2C: effect estimate of mediator to dependent variable; dotted line represents c path = adjusted for confounders and c' path = in addition to confounders also adjusted for mediator. Confounders = age, sex, alcohol consumption, body mass index, smoking status, maximum oxygen uptake, hypertension and diabetes status. Mediator = serum advanced oxidation protein product content. [file jgs0062-0222-SD1.docx]

**APPENDIX -** SUPPLEMENTARY MATERIALS FOR ONLINE ONLY

| **Table S1** Association between z(lnLTL) with Age, Sex and Cardiovascular Disease Risk Factors: Austrian Stroke Prevention Study | | | | | |
| --- | --- | --- | --- | --- | --- |
| Risk factors or  subclinical disease | Unstandardized  β coefficient^a^ | | 95% CI | | *p* value |
|  |  |  | LB | UB |  |
| Age | -.008 | | -.016 | .000 | .04 |
| Gender (female) | -.008 | | -.019 | .003 | .13 |
| Diabetes^b^ | -.018 | | -.105 | .070 | >.15 |
| Hypertensive^c^ | -.041 | | -.195 | .112 | >.15 |
| Ever smoker^d^ | .097 | | -.048 | .242 | >.15 |
| Cardiac disease^e^ | -.011 | | -.150 | .128 | >.15 |
| BMI (*kg/m^2^*)^f^ | .020 | | .004 | .036 | .02 |
| C-reactive protein(*mg/liter*) | | -.006 | -.025 | .013 | >.15 |

^a,^ adjusted for age and gender using multiple linear regression

^b^ diabetes baseline(0= no, 1= yes (history of diabetes or insulin fasting glucose ≥126 mg/dl or treatment))

^c^ hypertension baseline (0= no,1= yes (history of hypertension or ≥140/90 mm Hg))

^d^ smoking status (0= non-smoker,1= ever smoker (which includes current and former smoker))

^e^ cardiac disease (0= no, 1= if there was evidence of coronary heart disease according to the Rose questionnaire, or appropriate Electrocardiogram findings, or if an individual presented signs of left ventricular hypertrophy on echocardiogram, or atrial fibrillation diagnosed by ECG and peripheral artery disease was diagnosed based on history)

^f^ defined as body mass index (kg/m^2^) ≥25 for female and ≥27 for male

| **Table S2** Association of Anti-oxidative Micronutrients on Telomere Length, Mediated by Serum AOPP | | | |
| --- | --- | --- | --- |
|  | **Direct effects** | | **Indirect effects** |
|  | no Mediator^a^  Beta (c path) | adjusted for Mediator^b^  Beta (c’ path) | Proposed mediator: *AOPP*  β^c^, 95% CI (boot LL, boot UL) |
| **Individual micronutrient z-score(s)** | | | |
| lutein+zeaxanthin | .120 | .112 | .0002(-.0022,.0059) |
| β-Cryptoxanthin | .040 | .042 | .0001(-.0051,.0057) |
| canthaxanthin | .056 | .058 | -.0012(-.0131,.0031) |
| lycopene | -.069 | -.052 | .0005(-.0020,.0090) |
| α-carotene | -.028 | -.022 | -.0008(-.0089,.0029) |
| β-carotene | -.077 | -.053 | -.0012(-.0136,.0083) |
| α-tocopherol | .019 | .006 | .0027(-.0128,.0244) |
| γ-tocopherol | .044 | .009 | .0034(-.0158,.0332) |
| vitamin C | .146 | .181 | -.0005(-.0121,.0047) |
| retinol | -.058 | -.095 | .0051(-.0082,.0311) |
| **Sub-groups micronutrient z-score(s)** | | | |
| Provitamin A | -.033 | -.024 | -.0002(-.0040,.0012) |
| Non-provitamin A | .082 | .082 | -.0003(-.0046,.0013) |
| Vitamin E | .043 | .010 | .0028(-.0186,.0273) |
| **Total burden z-score** | .033 | .043 | .0001(-.0016,.0016) |

***^a^*** adjusted for age, sex, alcohol consumption, BMI, VO_2_max., smoking status, diabetes status and hypertension status

***^b^*** further adjusted for mediator AOPP (advanced oxidation protein product)

***^c^*** unstandardized beta-coefficients

**Figure S1**

**(B2)**

Mediator

βAB2 βB2C

**(A)**

Antioxidative Micronutrients

**(C)**

z(lnLTL)

Also adjusted for mediator (**c’ path**)

Adjusted for confounders (**c path**)

βAB1 βB1C

**(B1)**

Confounders
